# Editorial: Aquatic Invertebrate Immunity Against Infectious Diseases

**DOI:** 10.3389/fimmu.2021.762082

**Published:** 2021-09-22

**Authors:** Luciane Maria Perazzolo, Chaozheng Li, Kunlaya Somboonwiwat

**Affiliations:** ^1^ Laboratory of Immunology Applied to Aquaculture, Department of Cell Biology, Embryology and Genetics, Federal University of Santa Catarina, Florianópolis, Brazil; ^2^ Southern Marine Science and Engineering Guangdong Laboratory (Zhuhai)/ State Key Laboratory of Biocontrol, School of Marine Sciences, Sun Yat-Sen University, Guangzhou, China; ^3^ Center of Excellence for Molecular Biology and Genomics of Shrimp, Department of Biochemistry, Faculty of Science, Chulalongkorn University, Bangkok, Thailand

**Keywords:** farmed aquatic invertebrates, host-pathogen-microbiota interactions, antiviral immunity, antimicrobial defense, crustaceans, mollusks

Aquatic invertebrates farming represents an important trade in the food supply of the global economy. Despite the expanding commercial relevance of shellfish farming, emerging diseases have caused catastrophic losses by limiting aquaculture sustainability. Crustaceans and mollusks cultures face numerous challenges, notably viral, bacterial, and fungal infections. Invertebrates lack classical adaptive immunity and only rely on innate responses to protect them from pathogens. Shrimp, oysters, scallops, mussels, crayfish, and crabs are among the most valuable farmed invertebrates. Understanding interactions between its immunity and pathogens is critical to controlling farming diseases. In light of this, nowadays, research on shellfish immunity has received remarkable efforts to unravel the molecular mechanisms that drive the immune responses against pathogens and, ultimately, to reach new non-antibody-based therapies to protect both cultivated and wild species. This Research Topic compiles 13 articles, including original studies and reviews by renowned scientists and research groups, which integrate current progress and understanding regarding antiviral and antimicrobial defenses triggered by aquatic invertebrates to control infectious diseases.

## Pathogen Recognition and Antimicrobial Defenses

Like other invertebrates, shellfish possess cellular and humoral defenses to protect them against pathogens. However, defense responses must be tightly regulated to allow the destruction of pathogens without harming the host. In this Research Topic, Bouallegui reviews crayfish immunity, addressing plague disease, molecular aspects of hematopoiesis, and highlighting cell-mediated responses associated with Extracellular trap cell death (ETosis). During pathogen recognition, the host’s pattern recognition receptors/proteins (PRRs/PRPs) recognize and bind to microbial/pathogen-associated molecular patterns (MAMPs/PAMPs), inducing PAMP-triggered immunity (PTI). C-type lectins are crucial PRPs for immobilizing microorganisms by agglutination; shrimp lectins are also implicated in antibacterial and antiviral defenses (1). Qiu et al. describe a novel C-type lectin widely distributed in adult mud crab tissues, *Scylla paramamosain* (*Sp*CTL6), and discuss its immunoprotective effect against bacteria. In turn, Cao et al. report a new lectin expressed by the hepatopancreas of the red swamp crayfish (*Procambarus clarkii*) and discuss its agglutinating ability to promote bacterial clearance.

## Antiviral Immunity

In penaeid shrimp farming, selective breeding and biosecurity have become essential to prevent viral diseases based on the host’s robust resistance to specific pathogens (2). Both disease prevention and genetic improvement have been based on theoretical support for the interaction between host immunity and virus pathogenesis (3). In this Research Topic, Zhang et al. report and discuss that glycogen synthase kinase-3β (GSK3β) from shrimp *Litopenaeus vannamei* (*Lv*GSK3β) negatively regulates the activity of NF-κB pathway; RNAi mediated knockdown of *Lv*GSK3β raise the survival of the shrimp infected by White spot syndrome virus (WSSV). In turn, Li et al. reveal that the TRAF3 ortholog from the *L. vannamei *(*Lv*TRAF3) could protect shrimp against WSSV infection by mediating antiviral defense throughout the IRF-Vago pathway, but not by the NF-кB pathway. Altogether, these findings describe shrimp immune responses against WSSV and suggest potential candidate genes for resistance-based antiviral defense. Another relevant contribution is made by Liao et al. concerning the decapod iridescent virus 1 (DIV1), an emergent virus responsible for recent economic troubles in Asian shrimp farming (4). Comparative transcriptome analysis reveals that DIV1 could hijack the triosephosphate isomerase-like genes (LvTPI) to facilitate virus replication in shrimp (Liao et al.). Consequently, LvTPI gene silencing reduces shrimp mortality by inhibiting viral replication. This study provides a theoretical basis for the epidemiological investigation of DIV1 infection and may help prevent viral diseases in shrimp farming.

## Cell-Mediated Responses

Hemocytes represent the immunocompetent cells of crustaceans and mollusks, responsible for cell-mediated responses against invading microorganisms and parasites. Regarding oyster immunity, cell-mediated responses include infiltration, phagocytosis, extracellular trap cell death, encapsulation, immune genes induction, and effectors secretion (5). In light of this, Lu et al. report and discuss the alteration in some hemato-immunological parameters in oyster *Crassostrea hongkongensis* associated with gender and subpopulation in oysters under bacterial challenge. Estrada et al. evaluated and discuss the occurrence of programmed cell death (PCD), apoptosis and pyroptosis-like, in hemocytes from *Crassostrea gigas* triggered by marine toxins produced by microalgae and bacteria.

## Host-Pathogen and Microbiota Interactions

Recent studies have revealed exciting aspects of the immune defense triggered by aquatic invertebrates in response to strategies used by pathogens to circumvent them. Insights into host-pathogen and microbiota interactions in fighting invaders have only recently started to emerge. In line with this notion, Petton et al. review and shed light on how the polymicrobial and multifactorial factors operate in the Pacific oyster mortality syndrome (POMS), the most prevalent disease in *C. gigas*. The authors underline the effect of environmental culture conditions and the oyster microbiota imbalance (dysbiosis) in promoting the POMS pathogenesis. Bivalves are sessile filter feeders that live closely associated with large and diverse communities of microorganisms that form their microbiota. In light of this, González et al. provide new insights into the interaction between the immune responses of scallop *Argopecten purpuratus* and its microbiota. Scallop antimicrobial peptides and proteins are implicated in maintaining microbial homeostasis and are critical molecules in orchestrating host-microbiota interactions. In turn, aspects of the infection of Chinese crab*, Eriocheir sinensis* by the pathogenic yeast are described by Jiang et al., using proteomic analysis. The disease caused by *Metschnikowia bicuspidata* seems to induce some antimicrobial mechanisms (proPO activation system, ROS, and phagocytosis), while the fungal infection suppresses hemolymph coagulation and tissue regeneration.

## Host Susceptibility Versus Tolerance to Microbial and Viral Infections

As already mentioned, microbial and viral infections have impacted aquatic invertebrates farming, and selective breeding and biosecurity in farms have become essential to prevent diseases based on the host’s robust resistance to specific pathogens (2). Surprisingly, the molecular mechanisms that drive the susceptibility and tolerance of aquatic invertebrates against pathogens remain elusive. A study conducted by Mai et al. report molecular aspects of differential gene expression in susceptible and resistant strains of shrimp to acute hepatopancreatic necrosis disease (AHPND). A set of candidate genes is proposed to be involved in bacterial pathogenesis, and these findings may aid in breeding programs to select AHPND resistant/tolerant shrimp. In turn, Escobedo-Bonilla provides a broad overview of the viral interference phenomenon in cultivated crustacean species. Exclusion of superinfection, virus-virus interaction, host cell receptor blockade, viral co-infection, and current assays to assess viral interference are widely discussed. Ultimately, a heterologous virus interference mechanism is proposed as a natural strategy to control the incidence of viral diseases in shrimp farming.

## Concluding Remarks

This Research Topic brings new studies and insights into the molecular, cellular, and physiological mechanisms implicated in antimicrobial and antiviral defenses triggered by the aquatic invertebrates. Original articles and reviews provide novel sharpness into the intricate puzzle of pathogen-host interaction, the role of microbiota in host health and homeostasis, intracellular pathways driving immune effectors/regulators, viral interference phenomena, host susceptibility and tolerance to infections, and other issues. Elucidating the multifaceted mechanisms driving the immunity of farmed aquatic invertebrates is crucial to building new non-antibody-based therapies and controlling aquaculture diseases. Hopefully, the exciting outcomes displayed here can contribute to developing tools that increase shellfish immunocompetence and ultimately promote health security in aquaculture.

## Author Contributions

All authors co-edited the Research Topic. LMP substantially contributed to the conception, drafting, and editing of the final version of the Editorial. CL and KS contributed by drafting part of the work and approved it for publication. All authors contributed to the article and approved the submitted version.

## Conflict of Interest

The authors declare that the research was conducted in the absence of any commercial or financial relationships that could be construed as a potential conflict of interest.

## Publisher’s Note

All claims expressed in this article are solely those of the authors and do not necessarily represent those of their affiliated organizations, or those of the publisher, the editors and the reviewers. Any product that may be evaluated in this article, or claim that may be made by its manufacturer, is not guaranteed or endorsed by the publisher.
